# Editorial: Mitigating implicit bias and promoting compassionate behavior in public health/healthcare professionals: implications for treatment outcomes

**DOI:** 10.3389/fphar.2023.1306297

**Published:** 2023-10-18

**Authors:** Lon J. Van Winkle, Shane L. Rogers, Marta J. Brooks, Bianca B. Calderon, Nicole Michels

**Affiliations:** ^1^ Department of Medical Humanities, Rocky Vista University, Parker, CO, United States; ^2^ Department of Biochemistry, Midwestern University, Downers Grove, IL, United States; ^3^ School of Arts and Humanities, Edith Cowan University, Joondalup, WA, Australia; ^4^ School of Pharmacy, Regis University, Denver, CO, United States

**Keywords:** public health, critical reflection, compassion, implicit bias, life-long learning

## Introduction

Partly because it is unconscious, managing implicit bias in public health/healthcare remains intractable. Hence, administrators, practitioners, and their students unknowingly discriminate against others especially including socioeconomically disadvantaged people in the US and elsewhere. This discrimination leads to inferior patient-provider communication and associated interventions—such as medication treatment and adherence—as well as less desirable public healthcare systems more generally. Marketing of treatments to the public and recruitment of people for clinical trials are also influenced by implicit biases. Mitigation of these biases may, however, promote practitioner compassion and make them more cognizant of this discrimination. Hence, implicit bias mitigation should be incorporated into curricula for training all health administration/public health students, aspiring practitioners, and professionals themselves. Such an integral component of curricula likely would result in the life-long learning needed to foster better public health including the health of those against whom we, hopefully, no longer discriminate. Papers in this Research Topic consider the extent of these issues and their solutions including caregiver and student education.

## Extent of these problems in the US and elsewhere

In the US, the adverse public health impacts of anti-black racism render such racism a societal disease. In their contribution to this Research Topic, Eisape and Nogueira focus primarily on these issues in the US. However, the negative impacts of this treatment of others are, of course, not limited to the US (e.g., Patel et al., 2019; Narkowicz, 2023). In their paper, Eisape and Nogueira outline how racism adversely affects three “action spaces” to deliver inequitable disease outcomes to black people in the US. The first of these action spaces—disease governance—undervalues experiences of persons not living in the normalized white community. Consequently, people of color have higher prevalence of diseases, such COVID-19 infections, and are more likely to be hospitalized and die from these diseases and other disorders. In this regard, three governance interventions in China and Taiwan improved aspects of healthcare delivery in those nations, but it is unclear whether the interventions could be equitably employed in the US and elsewhere. The latter interventions are discussed further in three contributions to this Research Topic (Guo et al., Wen et al., Lin et al.).

According to Eisape and Nogueira, also contributing to poorer disease outcomes for black people in the US are two other action spaces—disease course and disease burden of black people. For instance, incomplete collection of data concerning the demographics of COVID-19 infections led to inadequate understanding of the course of this disease especially in Black populations. Similarly, during this pandemic, black people living in marginalized communities were usually unable to leave environments of high infection, thus, increasing their disease burden. These biases against people of color also likely contribute to serious—and systemically unconscious—inadequacies in the education of US healthcare professionals.

## Insufficient educational efforts for caregivers and their students

Efforts to educate healthcare professional students, and their clinical and basic science instructors, about the adverse effects of implicit bias on healthcare delivery also remain far too limited. For example, in North America, fewer than half of family medicine residencies consider systemic racism in their curricula (Bridges et al., 2023). And such shortcomings are glaringly evident to many medical students. For example, one student made the broader point that failure to recognize bulls-eye lesions on darker skin stems from the notion in medicine that “normal” skin is white (Nolen, 2020). To mitigate such erroneous notions and more, Freeman (2020) elegantly outlined four key ways to evaluate US medical schools, and such assessments should be implemented in pertinent ways in all healthcare professional curricula worldwide. According to Freeman, assessment of healthcare professional training should include whether the school 1) produces a graduate population that resemble the population of the country as a whole, 2) forces students to discover—in deeply personal ways—and then mitigate against harmful social determinants of health, 3) explicitly and actively works to counteract negative healthcare disparities, and 4) identifies—and identifies with—the community it serves and includes that community in decision making by the institution. The will to require schools to incorporate these recommendations into their healthcare curricula must be fostered in administrators, faculty, students, and members of communities. In turn, assessment by accrediting bodies should foster that will.

Two papers in the present Research Topic begin partially to address some of these needs. In their contribution to this Research Topic, Arif and Schlotfeldt describe how shortcomings in our efforts to measure and mitigate implicit biases in healthcare professionals and their students contribute to the issues needing better curricular assessments described above (Arif and Schlotfeldt). For example, the implicit association test is used widely to measure unconscious bias, but evidence for its value in constructing healthcare curricula is limited. Similarly, Arif and Schlotfeldt question whether recognizing implicit bias can sufficiently change healthcare professional’s behavior. In this regard, however, Van Winkle et al. report in this Research Topic that prospective medical students’ reported behavior can be improved at least temporarily through critical reflection on experiences serving their communities (Van Winkle et al.). Nevertheless, we contend that the curricular issues described above will persist as long as healthcare administrators and practitioners do not learn regularly and consistently to reflect critically and act on biases contributing to healthcare injustices. This reflection leading to improvements in curricula, and ultimately/ideally patient care, should continue throughout the careers of these medical professionals.

## Conclusion

While efforts to mitigate Implicit Bias and promote compassionate behavior in public health/healthcare professionals and their students have, so far, been too limited, there is reason for optimism. In their contribution to this Research Topic, Van Winkle et al. cite numerous papers showing that this bias can be mitigated in healthcare professional students even in basic science courses. They then report significant changes in students’ behavior that last at least as long as do their bias mitigating activities. Increasingly, and on their own, students are also becoming aware of their need to advocate for equity in the treatment of all patients (e.g., Nolen, 2020). With pressure to act accordingly, we believe accrediting bodies will adopt the assessment criteria outlined above (Freeman, 2020) in order to foster justice for all who need healthcare.
